# Comparative study of autonomic dysfunction between Parkinson’s disease with LRRK2, PRKN, and GBA mutations

**DOI:** 10.3389/fneur.2025.1657824

**Published:** 2025-08-19

**Authors:** Bárbara Maldotti Dalla Corte, Nayron Medeiros Soares, Eurípedes Gomes de Carvalho Neto, Carlos Roberto de Mello Rieder

**Affiliations:** ^1^Medical Sciences Postgraduate Program, Federal University of Rio Grande do Sul, Porto Alegre, Brazil; ^2^Federal University of Health Sciences of Porto Alegre, Porto Alegre, Brazil

**Keywords:** dysautonomia, Parkinson’s disease, SCOPA-AUT, genetic, autonomic

## Abstract

**Background:**

Autonomic symptoms are among the most important factors determining the quality of life in patients with Parkinson’s disease (PD). This study aimed to assess the profile of autonomic dysfunction symptoms in three groups of patients with genetic PD, carrying mutations in *GBA*, *LRRK2*, and *PRKN* genes, compared with subjects with sporadic PD.

**Methods:**

This case–control observational secondary analysis of prospectively collected data was performed on 742 patients (485 in the sporadic group, 165 in the LRRK2 group, 85 in the GBA group, and nine in the PRKN group). Autonomic symptoms were evaluated using the Scale for Outcomes in Parkinson’s Disease-Autonomic (SCOPA-AUT).

**Results:**

The GBA group exhibited more severe autonomic linsymptoms than the sporadic group, even after controlling for potential confounders such as disease duration and levodopa equivalent daily dose (linear regression B value = −4.668; Total SCOPA-AUT: *p* = 0.050; LEDD: *p* = 0.966; Disease Duration: *p* = 0.498). The LRRK2 group initially showed more autonomic symptoms, but this did not remain significant after adjustment for disease duration (*B* value = −3.105; *p* = 0.189). The PRKN group did not differ significantly from the sporadic group. Subgroup analysis highlighted specific issues including constipation, early satiety, and heat intolerance in both the GBA and LRRK2 groups, orthostatic hypotension in the GBA group and urinary incontinence and excessive perspiration in the LRRK2 group. Despite these subjective reports, objective assessment for orthostatic hypotension revealed no significant inter-group differences.

**Conclusion:**

These findings that genetic background may influence the severity of autonomic dysfunction in PD. In particular, patients with *GBA* mutations appear to experience a greater autonomic symptom burden, underscoring the need for personalized clinical monitoring and further research into genotype-specific disease progression. However, inconsistencies between subjective reports and objective autonomic measures emphasize the importance of employing more refined and sensitive assessment tools. Larger and demographically balanced cohorts are required to confirm these results, especially for the underpowered PRKN.

## 1 Introduction

Parkinson’s disease (PD) is one of the most common neurodegenerative disorders after Alzheimer’s dementia (1). The dopaminergic deficit owing to the loss of neurons in the substantia nigra pars compacta mostly leads to classic motor symptoms that are prominent and mandatory for the diagnosis of PD (2); however, when they become clinically apparent, approximately 50% of those neurons are already lost (3). This highlights the importance of non-motor symptoms, as mentioned in the first report of the 19th century, which appear as constipation, drooling, and rapid eye movement sleep behavior disorder (4). Non-motor symptoms are the main determinants of the quality of life and institutionalization of patients with PD. (5) Among the non-motor symptoms, autonomic nervous system dysfunction is highly prevalent, with reported rates ranging from 30–65% (6). This dysfunction can affect cardiovascular, gastrointestinal, urogenital, and thermoregulatory systems. Its prevalence varies with disease duration, significantly increasing within the first 5 years after diagnosis. In addition to the quality of life, dysautonomic symptoms are related to depression, impairment of independence for daily living activities (7), mortality, and a less favorable response to levodopa treatment, among others (8). The importance of non-motor symptoms resides not only in their impact, but also in their early appearance in the pathological process. In recent decades, they have come even more to light, including the observation of their presence many years before the motor symptoms and, hence, the clinical diagnosis and chance of treatment. This observation is consistent with the disruptive findings about the pathophysiology of PD described by Braak et al. (9), which led to the development of a list of markers for prodromal PD that are currently significant for research purposes (10). The discovery of genetic bases in the 1990s broadened the spectrum of the pathological mechanisms underlying dopaminergic and other neurotransmitter loss. In addition to monogenic forms of PD, which represent 5–10% of patients (11), many pathogenic variants with reduced penetrance or low effect sizes have been shown to increase the risk of PD in a non-Mendelian manner (12). In addition to the alpha-synuclein deposition pathophysiology, these genes (both monogenic and non-monogenic) act in different pathways such as mitochondrial dysfunction and neuroinflammation (13, 14). These findings led us to consider PD in a new way in which protein deposition is not the only pathogenic mechanism and raise questions about whether the genetic forms may be a different disease. Considering this perspective and the impact of dysautonomic symptoms, the main goal of this study was to assess differences in the magnitude of dysautonomic symptoms between patients with sporadic and genetic forms of PD, seeking more personalized care for our patients and new insights into their clinical and epidemiological profiles. There are three genes of special interest: the *PRKN* gene, located at 6q25.2–27 (15), which is related to mitochondrial maintenance (12) and the most common mutation of autosomal recessive disease (16); *LRRK2*, located at 12q12–q13.1 (17), is the most frequent cause of autosomal dominant disease and related to sporadic forms with age-dependent penetrance (16) and functions in transport and protein synthesis (12); and *GBA*, the gene that codes the enzyme glucocerebrosidase, which is essential to the sphingolipid degradation pathway (16). Mutations in *GBA* are the strongest known genetic risk factor for PD, though they do not follow Mendelian inheritance (12). Prior studies have linked *GBA* mutations to a higher risk of cognitive decline (18). More recently, clinical impressions and limited data have suggested that these mutations may also be associated with more severe autonomic dysfunction, particularly in the cardiovascular domain (19). In addition to assessing autonomic symptoms, this study also sought to characterize the clinical and sociodemographic profiles of patients with different genetic backgrounds.

## 2 Materials and methods

In this cross-sectional, observational, case–control study, data samples were obtained from the Parkinson’s Progression Markers Initiative (PPMI) with previously authorized access. The PPMI is a longitudinal, observational, multicenter, natural history study sponsored by the Michael J. Fox Foundation which assesses the progression of clinical features, imaging outcomes, and biological and genetic markers across stages of PD, from prodromal to moderate. Currently, it has approximately 4,000 participants enrolled at about 50 sites worldwide. The “case” group was composed of participants with an established diagnosis of PD with mutations in *PRKN*, *LRRK2*, or *GBA*. The “control” group was composed of participants with an established diagnosis of sporadic PD. The exclusion criterion was the absence of clinical criteria for PD, even if gene mutations were identified. The original study constituted a cohort. However, we performed a retrospective case–control analysis of the data collected at the enrollment assessment (between 2010 and 2022). The sociodemographic features addressed were age, sex assigned at birth, ethnicity, ancestry, and family history (history of PD or Parkinsonism in parents or grandparents). The clinical condition was assessed through variables such as disease duration, Hoehn and Yahr scale (20), and levodopa equivalent daily dose (LEDD) (21–23). The dysautonomic symptoms were specifically assessed using the Scale for Outcomes in Parkinson’s Disease-Autonomic (SCOPA-AUT) (24), which comprises questions evaluating gastrointestinal, urinary, cardiovascular, thermoregulatory, pupillomotor, and sexual dysfunctions. Additionally, specific questions of the Movement Disorder Society-Unified Parkinson’s disease Rating Scale (MDS-UPDRS) (25) that address those manifestations (1.10, 1.11, and 1.12), and objective measures of blood pressure, standardized for orthostatic hypotension evaluation (26), were utilized. Statistical tests were selected according to distribution using the Shapiro–Wilk test of normality. The quantitative variables included age, disease duration, LEDD, Hoehn and Yahr scale, SCOPA-AUT Scale, and the selected MDS-UPDRS questions. They exhibit a nonparametric distribution, and are shown using median, minimum, and maximum values. The data were analyzed using the Kruskal–Wallis test, and the methods for comparison between groups were Mann–Whitney, pairwise comparisons, and multiple linear regression with potential confounders controlled. Qualitative variables were analyzed using Chi-Square test, using the Pearson Chi-Square or Fisher’s Exact test as necessary, and shown in a frequency table; comparisons were made using the adjusted residual. The data handling occurred in RStudio 2022.12.0 for Windows and Excel Office 16 for Windows. The analysis was conducted in IBM SPSS Statistics 24, and graphic production was performed using GraphPad Prism 9.5.1.

## 3 Results

**Figure 1:**
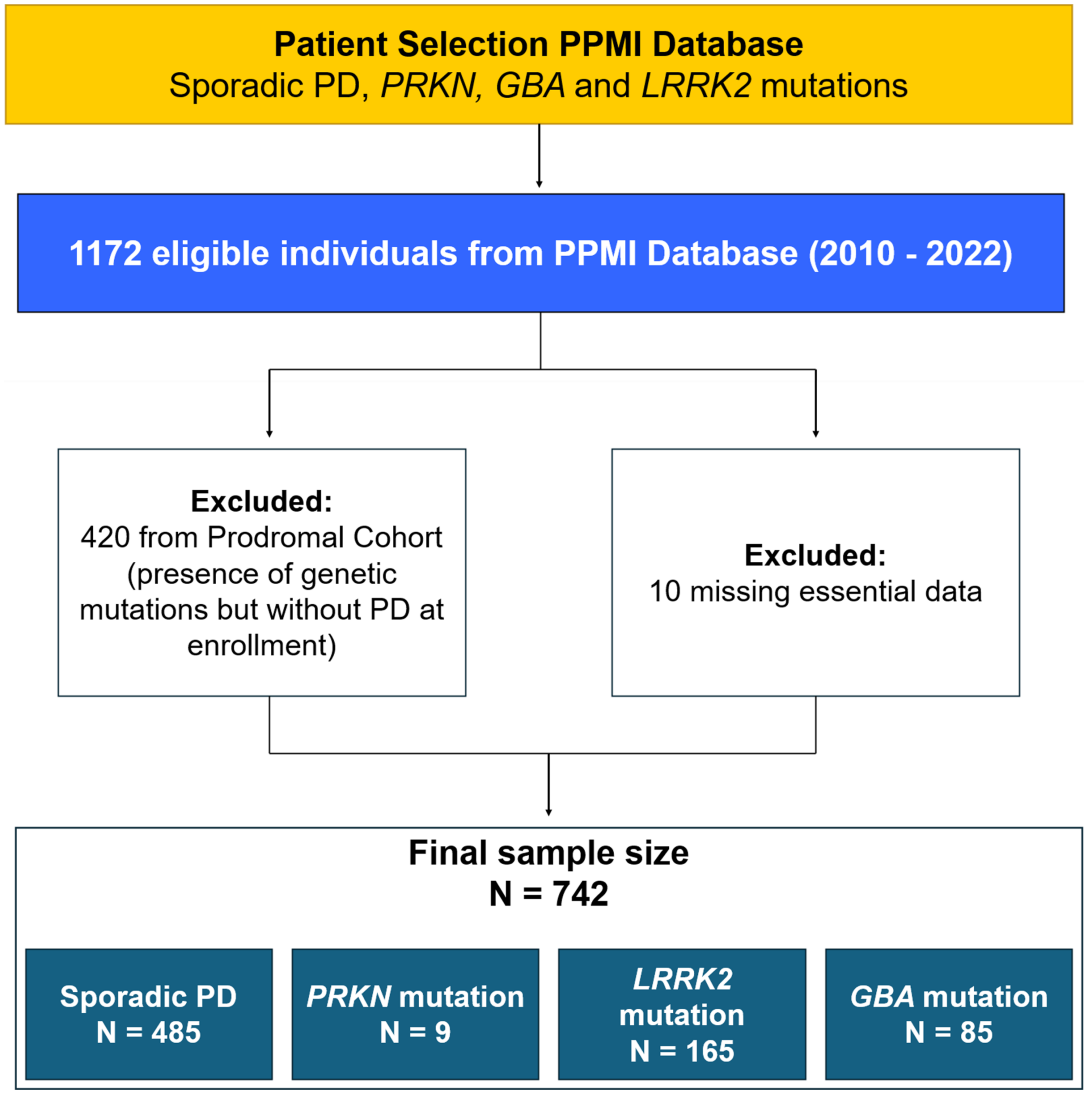
Flow diagram of patient selection.

**Figure 2:**
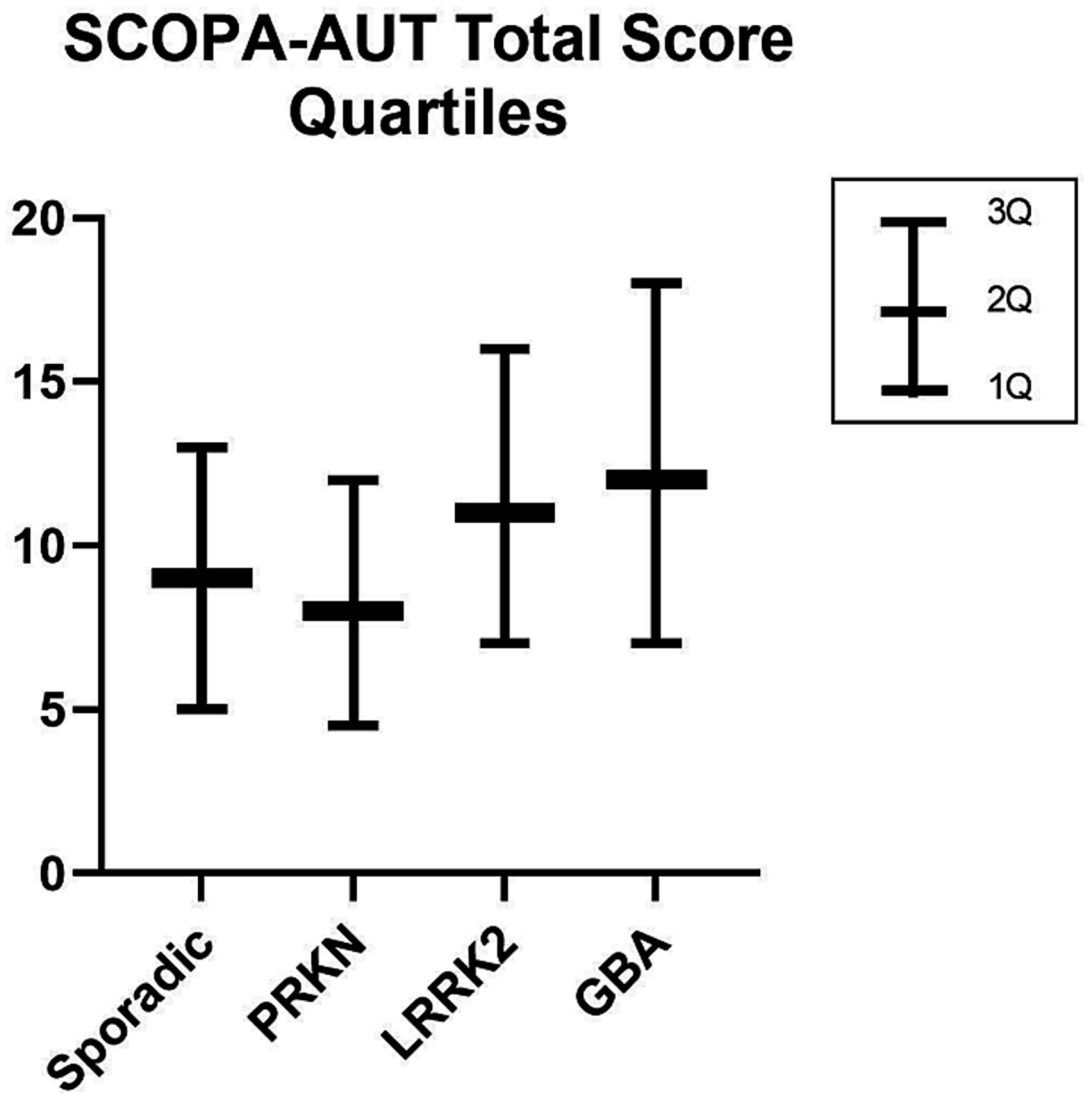
Quartile chart of SCOPA-AUT total score.

**Figure 3:**
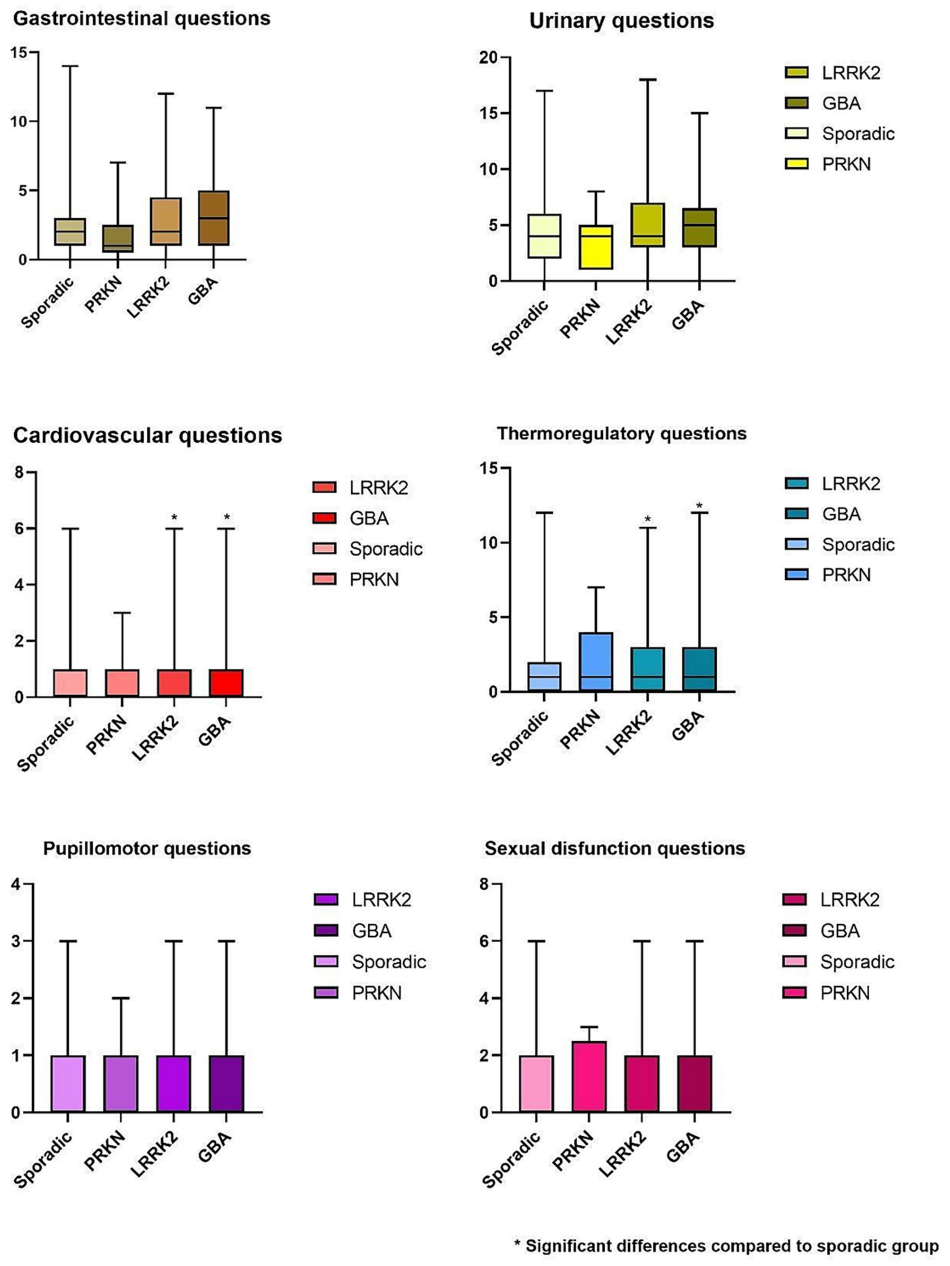
Sum of SCOPA-AUT by system.

There were 1,172 eligible individuals, 420 of whom were excluded because they had mutations in one of the genes of interest but did not meet the criteria for PD at enrollment (being followed in a prodromal cohort from the PPMI); 10 were excluded because of missing essential data. The final sample size was 742: 485 in the sporadic group, 165 in the LRRK2 group, 85 in the GBA group, and 9 in the PRKN group (two patients had both *GBA* and *LRRK2* mutations; Figure 1). Table 1 summarizes the main sociodemographic and clinical characteristics of the patients. This study revealed sociodemographic differences between the groups. The PRKN group showed no sex, ethnicity, ancestry, or age predominance. However, a predominance of concomitant maternal and paternal inheritance was present, consistent with the known autosomal recessive Mendelian pattern. The LRRK2 group did not show predominance in aspects of ethnicity and ancestry but exhibited a female predominance and older age. Compared to both the sporadic and the PRKN groups, the LRRK2 group had a predominance of maternal inheritance. The GBA group exhibited no sex, ethnicity, or age differences, but a predominance of Ashkenazi Jewish ancestry (either isolated or associated with Basque ancestry) was noted. In terms of inheritance patterns, a lack of family history was predominant, which aligns with its non-Mendelian pattern of inheritance. Regarding the clinical aspects, all genetic groups in this sample had a longer disease duration than the sporadic group; the Hoehn and Yahr score was significantly higher in the LRRK2 and GBA groups compared with the sporadic group. LEDD showed no significant difference across the four groups. The dysautonomic symptoms were assessed using the SCOPA-AUT, specific questions of the MDS-UPDRS scale that address the autonomic nervous system (regarding urinary symptoms, constipation, and lightheadedness), and measures of blood pressure. The total SCOPA-AUT yielded a significant Kruskal–Wallis test result (*p* < 0.001), indicating statistically significant inter-group differences. The pairwise comparison revealed that these differences were between the sporadic and LRRK2 groups (adjusted *p* = 0.002) and the sporadic and GBA groups (adjusted *p* = 0.004). Additionally, a linear regression compared total SCOPA-AUT scores in each genetic group with those in the sporadic group, controlling for potential confounders; the results are summarized in Table 2. The GBA group exhibited significantly more global autonomic symptoms on the SCOPA-AUT, even after controlling for disease duration and LEDD (B value = −4.668; Total SCOPA-AUT: *p* = 0.050; LEDD: *p* = 0.966; Disease Duration: *p* = 0.498). The LRRK2 group initially showed significantly more autonomic symptoms than the sporadic group; however, this significance was lost upon controlling for confounders, specifically disease duration (B value = −3.105; Total SCOPA-AUT: *p* = 0.189; LEDD: *p* = 0.134; Disease Duration: *p* = 0.039). The dysautonomic symptoms in the PRKN group were comparable to those in the sporadic group. The SCOPA-AUT total score data are shown in the quartile chart (Figure 2). Specifically analyzing the subgroups of symptoms, the GBA group exhibited more cardiovascular and thermoregulatory symptoms (*p* = 0.007 and 0.006, respectively) and the LRRK2 group showed more gastrointestinal, thermoregulatory, and cardiovascular manifestations (*p* < 0.007, < 0.001, and 0.011, respectively). Table 3 provides the results of these symptoms comparisons arranged by system, and Figure 3 displays the sum of each part of the scale by group. On analyzing individual SCOPA-AUT questions, some demonstrated significant differences in the Kruskal–Wallis test; these are exhibited in Table 4 and will be detailed hereafter. Questions with a non-significant Kruskal–Wallis test are also described in Table 4, along with their descriptive statistics. Early satiety, constipation, and heat intolerance were more frequent in both the GBA and LRRK2 groups compared to the sporadic group. Urinary incontinence and excessive perspiration were more frequent in the LRRK2 group than in the sporadic group. Excessive salivation was more frequent in the GBA group compared to the LRRK2 group. For questions regarding straining at defecation and classical orthostatic hypotension symptoms, the Kruskal–Wallis test showed a significant result (*p* = 0.019), but the pairwise comparison only demonstrated non-adjusted significance in the statistically significant range; all the adjusted significances were > 0.05. Regarding the MDS-UPDRS selected questions, orthostatic hypotension symptoms were more severe in the GBA group compared to the sporadic group. Urinary incontinence and constipation did not demonstrate differences between groups in the Kruskal–Wallis test; the results are presented in Table 4 along with their descriptive statistics. The PRKN group did not show significant influences in the dysautonomic symptoms when specifically analyzing the subgroups of symptoms, or the SCOPA-AUT, or the specific questions of the MDS-UPDRS. Objective signs of orthostatic hypotension, assessed through drops in blood pressure 1 to 3 min after standing from a supine position, revealed no significant differences between the genetic and sporadic groups (PRKN: *p* = 0.662; LRRK2: *p* = 0.118; GBA: *p* = 0.344).

**Table 1:** Sociodemographic and clinical features of subjects according to their genetic status.

Sociodemographic/clinical feature	Sporadic	GBA	LRRK2	PRKN	*p*-value
Women/Men	153/332	41/44	90/75	2/7	<0.001^**††^
Family history					
Father	50	13	26	1	0.004††‡‡§§
Mother	38	5	42	0	
Father/Mother	4	0	1	1	
No family history	135	43	71	3	
No data	258	24	25	4	
Ethnicity					
Asian	10	0	1	0	0.572
Black	7	1	0	0	
White	457	84	162	9	
Am Indian/Alaska	2	0	0	0	
No data	9	0	2	0	
Ancestry					
African Berber	0	0	2	0	0.001^‡‡^
Ashkenazi Jewish	37	34	72	0	
Basque	1	0	6	0	
Hispaninc/Latino	19	1	28	1	
AJ + HL	2	0	1	0	
AJ + B	0	2	0	0	
AJ + B + HL	0	0	1	0	
AB + HL	1	0	0	0	
No data	425	48	55	8	
Age at evaluation (years)	62.70 (33.70 –84.90)	64.60 (23.30 –80.60)	65.40 (33.70 –85.20)	56.30 (29.30 –78.80)	0.003^†‡^
Disease duration (years)	1.00 (0–4)	3.00 (0 –11)	3.00 (0–8)	4.00 (0 –11)	<0.001^†II*^
Hohen and Yahr	2.00 (1–3)	2.00 (0 –3)	2.00 (0–3)	2.00 (1 –2)	<0.001^†II^
LEDD	300 (80 –2,172)	600 (50 –3,082)	520 (52 –2,847)	400 (52 –1,050)	0.447

AJ, Ashkenazi Jewish; HL, Hispanic/Latino; B, Berber; AB, African Berber. ** Significant difference in the sporadic group; †† Significant difference in the LRRK2 group; ‡‡Significant difference in the GBA group; §§ Significant difference in the PRKN group; † Significant differences between the sporadic and LRRK2 groups; ‡ Significant differences between the PRKN and LRRK2 groups; II Significant differences between the sporadic and GBA groups; * Significant differences between the sporadic and PRKN groups.

**Table 2:** Total SCOPA-AUT values comparison between 289 genetic and sporadic groups using linear regression.

Variables	*B*	*p*
GBA*	−4.668	0.050
LRRK2*	−3.105	0.189
PRKN	0.306	0.892

*Controlled by LEDD and disease duration.

**Table 3:** Mann–Whitney p-values for dysautonomic features arranged by system for each genetic group of Parkinson’s disease compared with sporadic Parkinson’s disease group.

System	PRKN	LRRK2	GBA
Gastrointestinal	0.782	0.007	0.125
Urinary	0.413	0.092	0.152
Cardiovascular	0.507	0.011	0.007
Thermoregulatory	0.321	<0.001	0.006
Pupillomotor	0.892	0.413	0.234
Sexual disfunction	0.611	0.511	0.341

**Table 4:** Dysautonomic features of patients with Parkinson’s disease using SCOPA-AUT and MDS-UPDRS according to genetic status.

SCOPA-AUT/MDS-UPDRS question number	Sporadic	GBA	LRRK2	PRKN	*p*-value
Gastrointestinal					
SCAU1	0 (0–3)	0 (0–2)	0 (0–3)	0 (0–2)	0.731
SCAU 2	0 (0–3)	0 (0–3)	0 (0–3)	0 (0–1)	0.027*
SCAU 3	0 (0–3)	0 (0–2)	0 (0–3)	0 (0–2)	0.243
SCAU 4	0 (0–3)	0 (0–3)	0 (0–3)	0 (0–1)	<0.001^†II^
SCAU 5	0 (0–3)	0 (0–3)	0 (0–3)	0 (0–1)	<0.001^†II^
SCAU 6	1.00 (0–3)	1.00 (0 −3)	1.00 (0–3)	0 (0–1)	0.019
SCAU 7	0 (0–2)	0 (0–1)	0 (0–3)	0 (0–1)	0.571
Urinary					
SCAU 8	0 (0–3)	0 (0–3)	0 (0–3)	0 (0–1)	0.012^†^
SCAU 9	0 (0–3)	0 (0–3)	0 (0–3)	0 (0–1)	0.125
SCAU 10	0 (0–3)	1.00 (0 -3)	0 (0–3)	0 (0–2)	0.665
SCAU 11	0 (0–3)	0 (0–3)	0 (0–3)	0 (0–1)	0.972
SCAU 12	1.00 (0–3)	1.00 (0 −3)	1.00 (0–3)	1.00 (0 −2)	0.525
SCAU 13	1.00 (0–3)	1.00 (0 −3)	1.00 (0–3)	2.00 (0 −2)	0.789
Cardiovascular					
SCAU 14	0 (0–3)	0 (0–3)	0 (0–3)	0 (0–2)	0.019
SCAU 15	0 (0–3)	0 (0–3)	0 (0–3)	0 (0–1)	0.182
SCAU 16	0 (0–2)	0 (0–1)	0 (0–1)	0 (0–2)	0.561
Sudomotor					
SCAU 17	0 (0–3)	0 (0–3)	0 (0–3)	0 (0–2)	<0.001^†^
SCAU 18	0 (0–3)	0 (0–3)	0 (0–3)	0 (0–2)	<0.001^†^
SCAU 20	0 (0–3)	0 (0–3)	0 (0–3)	0 (0–3)	0.524
SCAU 21	0 (0–3)	0 (0–3)	0 (0–3)	0 (0–3)	<0.001^†II^
Pupillomotor					
SCAU 19	0 (0–3)	0 (0–3)	0 (0–3)	0 (0–2)	0.472
Genital - men					
SCAU 22	0 (0–3)	1.00 (0 −3)	0 (0–3)	0 (0–2)	0.234
SCAU 23	0 (0–3)	0 (0–3)	0 (0–3)	0 (0–2)	0.962
Genital - women					
SCAU 24	0 (0–3)	0 (0–3)	0 (0–3)	0 (0–3)	0.555
SCAU 25	0 (0–3)	0 (0–2)	1.00 (0–3)	1.00 (0 −3)	0.683
Total SCAU	9.00 (0–43)	12 (0 −29)	11.00 (0 −39)	8 (3–25)	<0.001^†II^
MDS 1.10 URIN	1.00 (0–4)	1.00 (0 −3)	0 (0–4)	0 (0–2)	0.288
MDS 1.11 CSTP	0 (0–4)	0 (0–4)	0 (0–3)	0 (0–1)	0.103
MDS 1.12 OH	0 (0–3)	0 (0–2)	0 (0–3)	0 (0–1)	0.012^II^

SCAU, question of the SCOPA-AUT; MDS, MDS-UPDRS scale; URIN, urinary symptoms; CSTP, constipation; OH, orthostatic hypotension. * Significant differences between the LRRK2 and GBA groups; † Significant differences between the sporadic and LRRK2 groups; II Significant differences between the sporadic and GBA groups.

## 4 Discussion

The differences in dysautonomic symptoms, more strongly evidenced in the GBA group, indicate a genetic influence on non-motor aspects of Parkinson’s disease and led us to think about it as a different disorder. As demonstrated in previous studies, the unbalanced function of glucocerebrosidase interferes with alpha-synuclein degradation, potentially leading to increased aggregation. Conversely, toxic alpha-synuclein aggregates reduce the lysosomal function, further accelerating protein aggregation (27). This pathogenic cellular loop may accelerate neurodegeneration and could explain the more severe manifestation of dysautonomic symptoms in patients with *GBA* mutations, observed even independent of disease duration. This enhances not only the need to explore this as a pathophysiological pathway, but also the importance of genetic testing, and individualized care of patients in daily practice. Special attention should be given to autonomic symptoms in patients with PD and *GBA* mutations. Although previous studies have reported significant cardiovascular dysautonomia in *GBA* mutation carriers (19), our study did not identify significant differences in objective measures of orthostatic hypotension, despite more prominent subjective symptoms in this group. This discrepancy may reflect the limited sensitivity of bedside assessments, such as manual blood pressure monitoring, when compared to more precise methods like the tilt-table testing or heart rate variability analysis (28). These findings underscore the need for systematic evaluation of autonomic function using dedicated instruments, particularly in genetic at-risk individuals. Early identification and management of autonomic dysfunction – ideally before overt symptom onset may improve quality of life and reduce cardiovascular risk. However, this study has limitations. First, the absence of more sensitive or diverse objective autonomic tests limits the interpretation of the findings. Second, the PRKN group was markedly underpowered (n = 9), precluding meaningful statistical comparisons. Despite that, more homogeneous groups in sociodemographic aspects would make the results stronger. Two other important aspects not approached in our study include the prodromal profiles of patients with these mutations and different variants of each gene. Our sample included at least five different variants in the GBA group and three variants in the LRRK2 group, and a separate analysis of them could demonstrate intragroup differences.

## 5 Conclusion

In summary, our findings suggest that patients with *GBA*-associated PD exhibit a greater burden of autonomic symptoms, independent of disease duration, supporting the role of genetic factors in the non-motor profile of the disease. The discrepancy between subjective complaints and objective autonomic measures highlights the need for more sensitive diagnostic tools. While the results for *LRRK2* were less robust after adjustment, and data on *PRKN* remain inconclusive due to the small sample size, our study reinforces the importance of considering genotype in the clinical evaluation of autonomic dysfunction in PD. Future research should explore prodromal features and variant-specific effects to improve personalized care.

## Data Availability

The datasets presented in this study can be found in online repositories. The names of the repository/repositories and accession number(s) can be found at: http://www.ppmi-info.org/access-data-specimens/download-data, RRID: SCR_006431.
